# Paying the Price for a Broken Healthcare System: Rethinking Employment, Labor, and Work in a Post-Pandemic World

**DOI:** 10.1177/0730888420923126

**Published:** 2020-08

**Authors:** Ariel C. Avgar, Adrienne E. Eaton, Rebecca Kolins Givan, Adam Seth Litwin

**Affiliations:** 1 ILR School, Cornell University, Ithaca, New York, USA; 2 School of Management and Labor Relations, Rutgers University, New Brunswick, New Jersey, USA

**Keywords:** healthcare, organizations, labor relations, professions, organizational behavior, technological change, Covid-19, unions

## Abstract

Even before the word *pandemic* reentered the lexicon, pressures stemming from institutional and technological change challenged policymakers and provider organizations to rethink core features of the manner in which we deliver healthcare. This essay introduces a special issue devoted to the consequences of change on the healthcare sector’s varied stakeholders. It does so in the context of our eventual, post-coronavirus reemergence and a renewed interest in remaking the healthcare system in light of its obvious deficiencies. Towards that end, we introduce the five papers composing this special issue, each of which informs the ways that change actually transpires in healthcare organizations and systems.

A pandemic of as yet unknown duration is changing the world. We do not know exactly how, but we can be certain this global crisis will upend governments and challenge established the social order. The ripples of the health crisis touch every aspect of society and every institution. Covid-19 exposes the inextricable relationship between access to healthcare, economic inequality, social stratification, global trade and migration, and the profound impact of public policy on who lives and who dies. While the edifice of healthcare provision may well be transformed, institutions, relationships, and path-dependent structures will determine precisely how this transpires and what system will emerge on the other side (Givan, 2016; Pierson, 1994).

This special issue features selected research from a conference cosponsored by Cornell and Rutgers Universities and held at the latter in January 2019—an era that will now surely be known as “Before the Pandemic.” As conference organizers, each of us had a firmly held belief in the significance of the study of healthcare as a window into broader questions in labor and employment relations and as a crucial sector in its own right. We, of course, expected to write an entirely different introduction to this issue—one that emphasized the centrality of the healthcare sector to industrialized economies and societies and the key insights it can provide regarding employment relations and work in other industries. But the world has changed swiftly and dramatically. Now, we need not persuade anyone of the importance of the organization and efficiency of healthcare systems. The Covid-19 crisis has placed near universal scrutiny on our healthcare systems, particularly public health and acute care organizations. The questions of how we lost our ability to prepare for, mitigate, or effectively respond to a pandemic that experts had long been predicting will doubtless inform research (and recriminations) for years to come. Along these lines, while the research presented in the following articles was completed before the Covid-19 pandemic arrived, it nonetheless sheds light on how healthcare organizations can and will respond to this unprecedented challenge. These papers all consider change either in healthcare organizations or in a healthcare system at a municipal level including technological and other organizational innovations and changes in labor relations. These workplaces are the point of delivery, where healthcare policies become actual healthcare, determining the health outcomes of individual patients and of society as a whole.

The common global crisis has illuminated the dramatic differences between healthcare systems across the world and the organizational and institutional factors that drive differences between these arrangements. Why has the U.S. healthcare system been so slow and ineffective in addressing many of the problems that are now playing out in full force? Payers, providers, and policymakers interact to create systems that organize and deliver healthcare. A national healthcare system is made up of massive, overlapping institutions, political priorities, professional and occupational workers and their attendant hierarchies, a vast administrative class, regulatory bodies, and insurers—all structured by embedded interests and incentives. This complex ecosystem underpins a set of workplaces, workers, and organizations that, at least in most cases, strive to achieve the often elusive “triple aim” of quality patient care for individuals, improved population health, and cost effectiveness (Berwick et al., 2008). In the current crisis, most Western healthcare systems fail to achieve all three of these aims. Insufficient resources preclude the delivery of quality patient care, and a lack of public health investment and preparedness erodes population health. In the case of the United States, longtime spending priorities are being exposed as devastatingly and lethally misguided.

The current crisis has highlighted a host of shortcomings in existing employment models, training, incentives, use of technology, supply chains, funding and investment, and so much more, perhaps most acutely felt in the United States. Nations are faced with a truly unprecedented opportunity to attend to and explain these shortcomings and consider how one might move from the current, fatally flawed system of healthcare access and delivery to one that works effectively and allows for a functioning, healthy society.

## U.S. Healthcare in the Age of the Novel Coronavirus

To say the U.S. healthcare sector wasn’t “designed” to respond to a pandemic would be too generous, as it implies at least a modicum of intentionality or perhaps some target objective aside from public health. And, as the late health economist Uwe Reinhardt (2013) quipped, no health policy expert would ever purposely conceive of our system for healthcare financing and delivery. He was referring to the multitude of overlapping yet insufficient funding methods and the fragmented, uncoordinated system for providing care, both of which contributed to the sluggish, unfeeling, and arguably ineffective response of “the system” to the outbreak.

The dysfunction first emerged on the demand side for healthcare. Initially by dint of historical accident—World War II price controls and Walter Reuther’s “Treaty of Detroit”—but then institutionalized by public policy—about half of Americans access health insurance through their employer or are dependent on someone who does (Kaiser Family Foundation, 2019). Consequently, the immediate economic impact of the pandemic and the government’s social distancing prescription was to disconnect 3.5 million people from their health coverage (Zipperer & Bivens, 2020).

Closer to the organizational and work-related issues we take up in this issue, however, rest the supply-side inadequacies of the system. To stay viable, payers and providers, public and private, strive to remain in the black. They do so by responding to a set of financial constraints and incentives that—while technically market-based—emerge from the long-term accretion of rules and regulations themselves heavily influenced by political interests.

One manifestation of this is providers’ orientation towards diagnosis and treatment. Under the dominant, “fee-for-service” model of healthcare financing, providers reap no economic benefit from disease prevention and little more from monitoring and managing their patients’ chronic diseases. We are not claiming that any particular clinician or frontline worker would ever consciously choose a cheaper, less effective course of action when confronted with an ailing patient. Much more likely, their employer has already made investments and structured care delivery in a way that precludes the most medically appropriate course of action. For example, a registered nurse’s daily schedule is already filled when she arrives at work in the morning. Without financial incentives, her employer is unlikely to reallocate a share of her time away from sick patients towards reaching out to otherwise well patients with long-term histories of congestive heart failure or type 2 diabetes. Imagine how little time she would have in her day to contact vulnerable but healthy patients to remind them to quarantine and explain how to monitor for Covid-19-related symptoms. Population health is disincentivized under the current system, and the pandemic has revealed the high death toll associated with this failing.

In much the same way, investments in public health—particularly by profit-maximizing corporate owners—put facilities at a competitive disadvantage. High-paying, privately-insured patients remain the most lucrative (Selden, 2020), explaining the increase in concierge hospital floors and the proliferation of expensive, but limited-use technologies such as surgical extenders like those considered by Menchik in this special issue. Likewise, hospitals seeking short-term savings often rely on contracted cleaners despite research showing that these workers are ill-trained and ill-equipped, with grave implications for the spread of infectious diseases much like Covid-19 (Litwin et al., 2017; Zuberi, 2013). Maintaining a workforce especially trained to manage pandemic levels of patient flow while maintaining their own safety represents a sizable, upfront cost with an uncertain and highly discounted future payoff. Likewise, an adequate supply of disposable, protective masks, ventilators, and bed capacity in preparation for a once-in-a-century, short-lived spike in demand makes little sense to nonclinician managers, let alone to shareholders (Rowland, 2020). Even at the pandemic’s apex, old-school jawboning more so than market forces pushed hospitals to boost capacity and to reorient themselves towards treating infected patients.

In actuality, many of the healthcare system’s shortcomings revealed during the pandemic have long existed and may persist even once we contain Covid-19 unless policymakers adopt meaningful reform. The sector’s languid embrace of new technologies that facilitate patient access without increasing costs or reducing quality serves as a case in point. While some raise reasonable concerns about the quality of care delivered telephonically, the comparison to conventional, in-person consultations seems inappropriate. Many of those opting to use telehealth services are patients who would not have made the investment of time and effort for a conventional appointment or simply could not find one of a shrinking number of primary care providers willing to see them. Yet, not until weeks into the pandemic did policymakers push insurers to start covering these telehealth encounters. Once they did, providers could use telehealth to ease the burden of low-acuity, non-Covid-19 patients and to keep those with coronavirus symptoms at a safe distance.

Until payers committed to reimbursing for telemedicine, uptake was minimal. It was mainly limited to those providers operating not under fee-for-service, but under the value-based care model, designed to reward quality rather than quantity of care. The latter could internalize the benefits of telehealth to better serve patients, including improved monitoring and management of their chronic diseases. As the current crisis begins to fade, providers, payers, and patients may be unwilling to give it up. One can only hope that worthwhile organizational and technological innovations forced by the coronavirus could have some staying power.

## The Legacy of the Low Road

From the perspective of work and organizations, the most glaring and longstanding shortcoming exposed by the crisis is the prevalence of low-road employment models applied to many frontline workers but especially to direct care workers, including home health aides, personal care assistants, emergency medical technicians, and certified nursing assistants. While physicians and nurses are often treated as core healthcare providers, many other staff have been treated by their employers—institutionally reified by payment systems—as ancillary and ripe for degradation and outsourcing in the name of cost savings and flexibility. Empirical evidence links the working conditions of frontline workers to the quality of care they provide (Aiken et al., 2010; Ochsner et al., 2009; Zuberi, 2013). Healthcare organizations that commit to quality frontline care jobs, for both nurses and support staff, that include good pay, benefits, job security, opportunities for advancement, and the ability to exercise meaningful voice, report better quality of care outcomes (Kochan et al., 2009). Nevertheless, far too many healthcare organizations in the United States make use of employment practices that rely on temporary, contingent, and low-wage arrangements. As such, direct care workers, who are disproportionately foreign-born women of color, are often outsourced, work multiple part-time jobs, and struggle to earn a living wage. Workers lacking basic employment protections are limited in their ability to properly care for society’s weakest and most vulnerable (Franzosa et al., 2019). These working conditions also make it difficult for employers to retain a stable, committed, and skilled workforce. It should come as no surprise to policymakers and to the healthcare executives now struggling to fill positions necessary to care for Covid-19 patients that decades of neglect of frontline worker jobs is one of the driving factors contributing to the shortage.

A hallmark of the prevalent low-road approach to the employment of direct care workers is minimal investments in training and skill development. Healthcare organizations are reluctant to invest training resources in a workforce that is viewed as easily replaceable and ancillary to their core mission. This, of course, is a short-sighted approach and comes at a substantial cost given the role that these workers do, in fact, play in the care of patients. Low-wage, direct care workers spend a substantial amount of time with patients, far exceeding that of physicians and nurses. With proper training, these workers could serve as an important part of the care team, increasing patient adherence to their care plan and identifying potential causes for concern. With a healthcare system struggling to meet the workforce demands it faces as a result of Covid-19, longstanding underinvestment in direct care worker training will likely jeopardize the health and safety of workers and patients alike.

To address the low-wage direct care worker shortage, we will need to restructure this work entirely. For instance, what if instead of assuming that home care workers lack skills and abilities, they were trained and challenged to become the lynchpin of care coordination for their clients? As Wu’s paper in this volume portends, tablets and smartphones could be deployed to provide them with a real-time portal for entering and extracting information about the patient and for coordinating with all of the patient’s providers, including specialists and social workers. A substantial pay increase would need to accompany the upskilling and increased centrality of these jobs, a needed change that would be hampered by current payment rules and low government-regulated payment levels.

As direct care workers put their lives at risk to help stem the spread of the Coronavirus, this is an especially fitting time to revisit previous calls for a new employment model that recognizes, rewards, and respects their contribution to our health, safety, and quality of life. In particular, we propose a model that includes four key features. First, direct care workers should be afforded working conditions that allow them to both care for their patients and support their families. Direct care jobs should, at the very least, provide a living wage and benefits, including paid sick leave, health insurance, and retirement contributions. These substandard jobs are disproportionately held by women; recent research shows that over one third of female healthcare workers earn under $15 per hour, with 17% of these workers either uninsured or covered by Medicaid (Himmelstein & Venkataramani, 2019). Second, healthcare organizations should move away from an approach that relies on temporary and contingent workers. Employers should, instead, commit to direct employment of the frontline workers and abandon the temptation of outsourcing these jobs to the lowest bidder. Our own research has demonstrated the clinical price that healthcare organizations pay for this approach (Litwin et al., 2017). Third, investments must be made in the formal and informal training and skill development of this workforce. Healthcare organizations should enhance their capacity to provide upskilling opportunities through mentoring programs and other informal training opportunities. Finally, direct care workers should have access to institutionalized mechanisms to speak up, exercise voice, and share their input. We know that doing so is associated with a host of benefits, including decreased turnover, increased commitment, and improved quality of care. Taken together, a high-road employment model consisting of these features will deliver gains to patients, workers, and the organizations that employ them. Employment relations researchers have documented the various benefits associated with high-road employment models. Now, Covid-19 is painting a painful portrait of the costs associated with the all too prevalent low-road models.

## Insights From the Papers Herein

In this moment, as researchers, we sit at home watching the healthcare system from a somewhat distanced vantage point. The commentary above is largely about that system. None of us, thankfully, are in a hospital, although we are all avid consumers of reports from the frontline, mostly mediated through journalism. This is an important point because the articles in this special issue are largely focused on the workplace. Four of the articles focus on changes taking place in specific healthcare workplaces. Two of these four papers deal with technological change or innovation though in very different contexts. As alluded to above, Menchik focuses on doctors’ (i.e., cardiologists’) adoption of new technology for performing ablations to treat arrhythmias, a robotic technology that mediates between the doctors’ hands and the patient. He develops a two-factor typology of individual approaches to adopting the new technology focused at one axis on the degree to which a doctor is influenced by their initial training and at the other, the influence from current colleagues. Also examining the link between work and technology, Wu compares the use of paper records to tablet computers for recording data on patient encounters by home health aides. She focuses on the unintended consequences that the technological shift has for the role of managers. Managers under the paper system mediated between the paper records created by direct care providers and the institutions’ official records; those under the tablet system are reduced to teaching and exhorting direct care workers to properly use their tablets.

Wu typifies the approach required to understand the workplace impact of new technologies in healthcare. The sector’s idiosyncratic structures and processes, not to mention its convoluted and complex work structures, nearly ensure that technology alone will not address deep-seated issues. While digital communications and artificial intelligence might make it easier for homebound patients to replenish their refrigerators or summon an ambulance (Osterman, 2017), the realities of home-based care make it unlikely that robots will “take care of grandma” anytime soon (Jacobs, 2018).

The other two workplace-focused papers also present important findings on occupational roles but in the context of different types of change. VanHeuvelen and Grace examine the move from a multi-occupancy ward design to single-patient rooms. They find that the four occupational/professional groups (i.e., registered nurses, respiratory therapists, nurse practitioners, and physicians) involved differed in their embrace or resistance to the change with some groups focused on the impact on patient care and others more on changes to working conditions. These initial attitudes were alleviated in some cases and confirmed in others after the implementation of the change. Wiedner, Nigam, and Bento da Silva examine an explicit attempt to change occupational roles in the English National Health Service, namely, by shifting elements of budgetary responsibility from career managers to physicians. Even in this very different healthcare system context, they, too, find that tensions among occupational groups, rooted in their differing “dispositions,” can derail an attempt at change.

All four of these papers point to the need to attend to the creation and subjectivity of occupational roles and occupational or professional identity in employer-driven projects of intentional change. The types of organizational changes discussed in these papers are likely to differ in significant ways from what is currently happening in healthcare institutions as this introduction is being written and Covid-19 cases are overwhelming hospitals in New York City. We know that Covid-19 has forced hospitals and long-term care providers in institutions or in client homes into changing practices dramatically and with little preparation. For instance, the city’s overwhelmed hospitals have effectively jettisoned protocols on changing personal protective equipment between patients and that masks and face shields intended to be used once are being reused after newly-invented and untested disinfecting routines. Hospitals are repurposing physical space in order to house more patients and attempting to isolate Covid-19 patients in spaces not meant for infection control (Hopkins, 2020). We cannot be sure that the nature of the rapid and forced changes taking place in real time as we write this introduction resembles the kind of change written about in this special issue.

From a purely intellectual view, it will be fascinating to see whether the same occupational dynamics at work in these papers are at play in this moment of crisis. Alternatively, some of the tensions around hierarchy and the policing of professional boundaries may get tossed aside when every healthcare worker is facing common fears about their own safety and the same heavy rates of patient mortality attendant to an overwhelmed system. More practically, while the potential for healthcare employers to have learned from these papers on how to better implement change under these conditions is somewhat doubtful, we do hope there are lessons for the longer run in the aftermath of the virus.

We turn finally to the fifth paper in this issue. Batt, Kallas and Appelbaum focus on a different level of analysis: the city, or more precisely, the healthcare system at the city level. The authors compare the labor relations approaches by employers in the healthcare systems in the upstate New York cities of Rochester and Buffalo. They argue that the different historical paths of industrial development in each city, which the paper traces, determined very different labor relations approaches with one emphasizing a more positive, cooperative stance by management and the other a more anti-union and hostile one. In turn, they argue these approaches determine the different paths healthcare employers in each city took in recent restructuring in response to similar external pressures and the ability of unions to respond to employer actions. It is clear at this moment that the city is an excellent level on which to focus our attention as the rate of infection and the strength of the healthcare system is, as a practical matter, focused in geographic areas. Batt et al.’s paper also provides a much-needed analysis of the possibilities for the exercise of worker voice through unionization, something that we can observe in real time in the middle of this crisis (Sengupta, 2020). It will be an interesting research question with strong practical application whether and how union representation makes a difference in outcomes for workers and for patients. In particular, we will be interested to see if or how the dynamics discussed in this paper play out in the upstate New York cities studied.^1^

We will never know whether a more intentionally and purposefully built U.S. healthcare system or even a more high-road version of the present one would have responded more effectively to Covid-19. Nonetheless, as we rebuild our system for “After the Pandemic,” we do hope the careful analyses gathered here will inform meaningful efforts at reform and improvement.

As we face the Covid-19 pandemic, so much uncertainty remains. What is certain, though, is that healthcare systems and organizations must change. The deeply-researched articles that follow suggest the most appropriate direction of change. Centering the needs of employees generally and more specifically employee voice and professional expertise, quality care, and investment in healthcare provision itself rather than bureaucracy or administration related to payment systems will be critical. Healthcare systems across the industrialized world will be shaken to the core by this crisis. When the time comes to rebuild, we will need to learn from the employment dynamics, relationships, and organizational structures of the “Before” era that exacerbated the toll of the pandemic. We must resolve to build a better system, with both “health” and “care” as its guideposts.

## Note

1 At the time of this writing there are relatively few cases reported in either Buffalo or Rochester.
